# Modelling and Realization of a Water-Gated Field Effect Transistor (WG-FET) Using 16-nm-Thick Mono-Si Film

**DOI:** 10.1038/s41598-017-12439-8

**Published:** 2017-09-22

**Authors:** Bedri Gurkan Sonmez, Ozan Ertop, Senol Mutlu

**Affiliations:** Department of Electrical and Electronics Engineering, Bogazici University, Istanbul, 34342 Turkey

## Abstract

We introduced a novel water-gated field effect transistor (WG-FET) which uses 16-nm-thick mono-Si film as active layer. WG-FET devices use electrical double layer (EDL) as gate insulator and operate under 1 V without causing any electrochemical reactions. Performance parameters based on voltage distribution on EDL are extracted and current-voltage relations are modelled. Both probe- and planar-gate WG-FETs with insulated and uninsulated source-drain electrodes are simulated, fabricated and tested. Best on/off ratios are measured for probe-gate devices as 23,000 A/A and 85,000 A/A with insulated and uninsulated source-drain electrodes, respectively. Planar-gate devices with source-drain insulation had inferior on/off ratio of 1,100 A/A with 600 μm gate distance and it decreased to 45 A/A when gate distance is increased to 3000 μm. Without source-drain electrode insulation, proper transistor operation is not obtained with planar-gate devices. All measurement results were in agreement with theoretical models. WG-FET is a promising device platform for microfluidic applications where sensors and read-out circuits can be integrated at transistor level.

## Introduction

By exploiting electrical double layer (EDL) concept and using electrical double layer capacitance (EDLC) as gate insulator, high channel control can be achieved at low gate voltages in field effect transistors (FETs)^1,2^. This provides a cheap solution to technologically and economically challenging problem of realizing an insulating layer as thin as possible without variations and pinholes. This phenomenon is explained by the combined Gouy-Chapman-Stern model^3^. Ionic liquids, ion-gels, aqueous and solid electrolytes are used to form EDL in these devices. They can use metal oxide semiconductors^4–6^, graphene^7–9^, carbon nanotubes^10–12^, organic semiconductors^13–15^ or Si^16–18^ as active channel layer.

Water as electrolyte material was used in organic field-effect transistors (OFETs) with probe-gate before^19^. Our group contributed to this concept by realizing a water-gated OFET with a planar-gate structure^20^. Planar-gate topology allows patterning of source, drain and gate electrodes on the same layer with a single photolithography step. It reduces fabrication process complexity and provides easy integration with fluidic channels. However, organic semiconductors are prone to ion diffusion which results in electrochemical doping^21^. Low charge carrier mobility and degradation due to environmental instability are other drawbacks of OFET devices.

**Figure 1:**
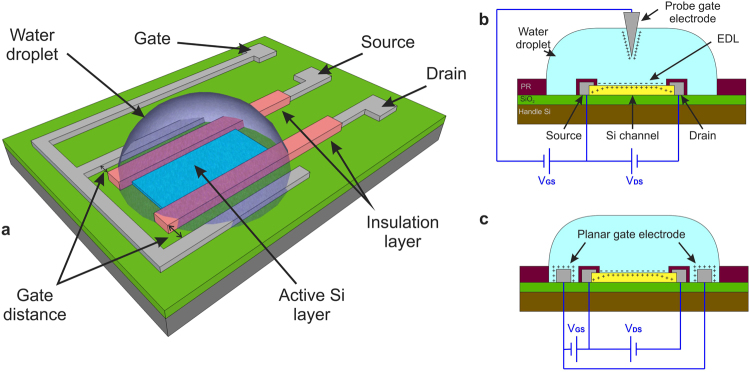
(**a**) An illustration of a WG-FET device with source-drain electrode insulation. Source, drain and gate electrodes are made of Al. A water droplet is placed on top of the Si active area to complete the device. Source and drain electrodes are insulated from water contact. Working principle of the WG-FET device is given for (**b**) probe and (**c**) planar gate setups. A negative voltage is applied to gate electrode to turn the transistor on.

In this paper, we present the realization and modelling of a water-gated field effect transistor (WG-FET) which uses 16-nm-thick mono-Si film as channel layer. A sketch of the WG-FET device is given in Fig. 1a. It combines the advantages of planar electrolyte-gated OFET design with the high performance of single crystalline Si layer.

Fluidic interfaces of these devices provide an integration platform for sensors and their read-out circuits at transistor level. Ultra-thin and high mobility characteristics of the channel layer provide better surface sensitivity in sensor applications and *in-situ* amplification in their read-out circuits. Two-dimensional electron gas (2DEG) feature of 16-nm-thick Si layer is exploited to obtain both in the same device^22,23^. Si nanowire FETs^24,25^ use similar concepts, however they use Si layers thicker than 25 nm for channel region and have more complicated fabrication steps. Label-free electronic sensors utilizing 30-nm-thick single crystalline Si layers have been shown as promising sensors^26^ but their usage as transistor or read-out circuit are not considered. Ion-sensitive FETs (ISFETs)^27–29^ resemble to these devices, however, they are bulk Si devices operating in depletion/inversion modes with large reference electrodes, and SiO_2_, Si_3_N_4_, Al_2_O_3_, Ta_2_O_5_ or other types of insulation layers. WG-FETs require simpler fabrication steps compared to these devices. Furthermore, neither planar and probe gate electrode topologies nor the effect of source and drain electrode insulation have been investigated and modelled before.

Working principle of WG-FET device is summarized for probe- and planar-gate topologies in Fig. 1b,c, respectively. Thin Si layer is moderately doped with Boron (~10^15^ cm^−3^). The WG-FET presented here is an accumulation type device. EDLs are formed on both gate electrode surface and Si active area. Positive charge carriers are attracted to the interface surface in Si layer with application of negative V_GS_ to the gate electrode. This turns on the transistor. For a thin Si film based accumulation mode p-channel MOSFET, expression of channel current for linear regime (*V*_DS_ > *V*_GS_ − *V*_FB_) is given as

1 $${I}_{{\rm{acc}}}=\frac{W}{L}{\mu }_{s}{C}_{{\rm{ox}}}[({V}_{{\rm{GS}}}-{V}_{{\rm{FB}}}){V}_{{\rm{DS}}}-\frac{{V}_{{\rm{DS}}}^{2}}{2}]$$

where *μ*_s_ is the hole surface mobility, *C*_ox_ is the oxide capacitance, *V*_GS_ is the applied gate voltage and *V*_DS_ is the drain-source voltage difference. *V*_FB_ is the flat-band voltage and it represents the threshold voltage for accumulation mode transistor since inversion does not occur^30^. The equation becomes

2 $${I}_{{\rm{acc}}}=\frac{W}{L}{\mu }_{s}\frac{{C}_{{\rm{ox}}}}{2}{({V}_{{\rm{GS}}}-{V}_{{\rm{FB}}})}^{2}$$

in saturation regime (*V*_DS_ ≤ *V*_GS_ − *V*_FB_).

In these equations, it is assumed that potential applied on the insulator layer throughout the channel is uniform and equal to *V*_GS_ which is the case for traditional MOSFET architecture. In WG-FET topology, effective *V*_GS_ is the voltage on the EDL which is formed on top of the active Si area. The value of this potential on any arbitrary point along the length of the device, *V*_g_EDL_(*x*), is a result of combined effects of source, drain and gate electrodes. Insulation on source and drain electrodes greatly reduces their effects on *V*_g_EDL_ by introducing extra serial capacitances with low values. Whether being insulated or not, their effects should be taken into account in calculating the channel current to obtain a more realistic model. Therefore, if the channel length is *L*, voltage of a point on EDL insulation layer can be written in most general form as

3 $${V}_{g\_\mathrm{EDL}}(x)={k}_{1}{V}_{{\rm{GS}}}+{k}_{2}{V}_{{\rm{DS}}}+({k}_{3}{V}_{{\rm{DS}}}+{k}_{4}{V}_{{\rm{GS}}})\frac{x}{L}$$

where *x* is from 0 to *L*. In probe-gate setup, gate electrode is placed in the middle of the channel, on top of the active area. Therefore, its effect is symmetric throughout the channel length. In planar-gate setup, gate electrode is designed surrounding the active area. Symmetric design ensures equal gate-source and gate-drain distances as in Fig. 1a. These symmetric effects of the gate electrodes result in an approximately uniform potential distribution throughout the channel, so the contribution of *V*_GS_ in *V*_g_EDL_ should not depend on *x* which makes *k*_4_ = 0.

To obtain an expression for the channel current, we can use the equation

4 $$I{\int }_{0}^{L}{\rm{d}}x=W{\mu }_{s}{C}_{{\rm{EDL}}}{\int }_{V\mathrm{(0)}}^{V(L)}[{V}_{g\_\mathrm{EDL}}(x)-{V}_{{\rm{thc}}}-V(x)]{\rm{d}}V$$

where *C*_EDL_ is the capacitance of EDL. *V*_thc_ stands for a threshold constant. It represents the cumulative effects of trapped charges at the Si/water interface with the flat-band voltage needed to form the channel in accumulation mode. If we use gradual channel approximation as $V(x)=({V}_{{\rm{DS}}}/L)x$ and substitute d*V* with $({V}_{{\rm{DS}}}/L){\rm{d}}x$, the channel current expression becomes

5 $$I{\int }_{0}^{L}{\rm{d}}x=W{\mu }_{s}{C}_{{\rm{EDL}}}{\int }_{0}^{L}[{V}_{g\_\mathrm{EDL}}(x)-{V}_{{\rm{thc}}}-\frac{{V}_{{\rm{DS}}}}{L}x]\frac{{V}_{{\rm{DS}}}}{L}{\rm{d}}x\mathrm{.}$$

If *V*_g_EDL_ (*x*) is replaced with the corresponding voltage distribution function, channel current can be found as

6 $$I=\frac{W}{L}{\mu }_{s}{C}_{{\rm{EDL}}}[({k}_{1}{V}_{{\rm{GS}}}+{k}_{2}{V}_{{\rm{DS}}}-{V}_{{\rm{thc}}}){V}_{{\rm{DS}}}-\frac{1-{k}_{3}}{2}{V}_{{\rm{DS}}}^{2}]$$

and in saturation it becomes

7 $${I}_{{\rm{sat}}}=\frac{({k}_{3}+1)W}{2L}{\mu }_{s}{C}_{{\rm{EDL}}}{({k}_{1}{V}_{{\rm{GS}}}+{k}_{2}{V}_{{\rm{DS}}}-{V}_{{\rm{thc}}})}^{2}\mathrm{.}$$

In *I*_sat_ expression of equation (7), *k*_1_ represents the gating capability of the applied *V*_GS_. *k*_2_ models the effect of drain electrode voltage on gating. When a negative *V*_DS_ is applied, it affects the transistor as a competing gate electrode. Therefore, it is desirable to have higher *k*_1_ and lower *k*_2_ values. *k*_3_ depends on both the effect of drain electrode and channel length. It acts as a common multiplier.

## Results

### Simulation Results

To verify the *V*_g_EDL_ (*x*) function and extract parameters, electric field and voltage distribution simulations of WG-FET devices with probe- and planar-gate setups are performed with COMSOL Multiphysics 5.2. For planar-gate setup, two symmetric gate electrodes are placed with a specific distance away from the source and drain electrodes. Two different layouts with gate electrode distances of 600 *μ*m and 3000 *μ*m are used to see the effect of planar gate electrode position on *V*_g_EDL_ (*x*). For each topology, versions with and without source-drain electrode insulation are simulated.

**Figure 2:**
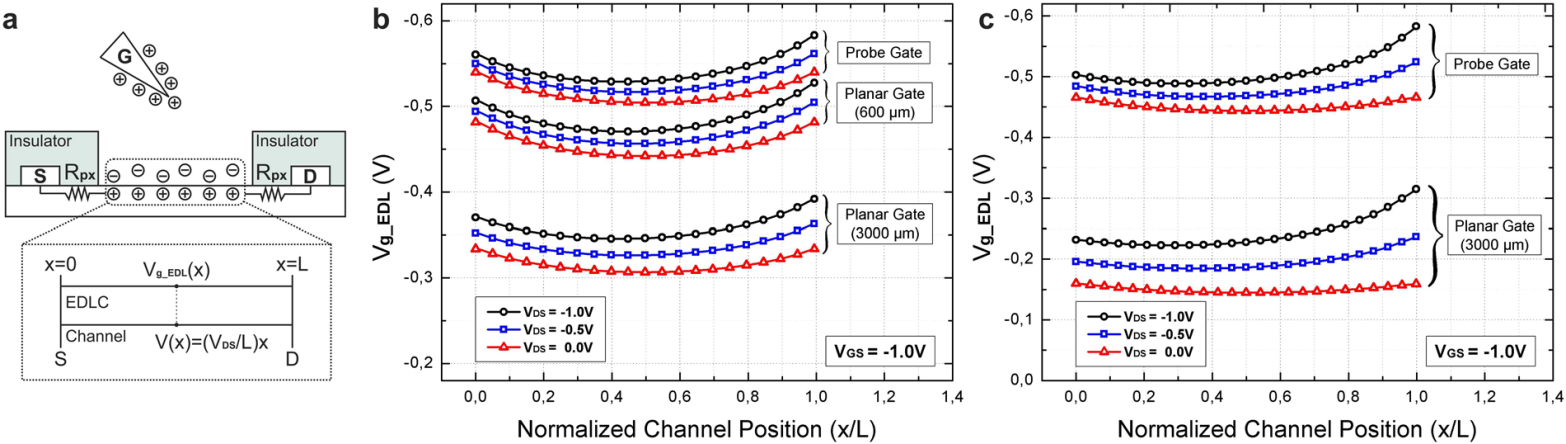
COMSOL Multiphysics 5.2 simulations of voltage distribution on EDL. (**a**) Device model used for simulations. *R*_px_ stands for the series parasitic resistance due to insulation overlapping. *V*_g_EDL_(*x*) vs. normalized channel position graphs for probe- and planar-gate setups with source-drain electrode insulation (**b**), and for devices with uninsulated source-drain electrodes (**c**).

Device model used in simulations for WG-FETs with source-drain electrode insulation is given in Fig. 2a. Due to alignment errors, insulating layer, photoresist (PR) in this case, overlaps with channel area which creates covered regions. These regions cannot be controlled by gate, therefore they behave like series parasitic resistances, *R*_px_, to the transistor. Value of *R*_px_ depends on fabrication process and can vary from sample to sample. In the case of devices with insulated source-drain electrodes, these parasitic effects are simulated using HSPICE as explained in the next section.

In simulations, voltage distribution on EDL is analyzed for different *V*_GS_ and *V*_DS_ values. Fig. 2b shows the simulation results for devices with source-drain electrode insulation. It can be seen that combined effects of drain and gate electrodes result in an uneven voltage distribution on EDL throughout the channel. In planar-gate setup with the closer gate electrode layout, effect of gate electrode on voltage distribution is lower compared to the probe-gate setup and it decreases further when the planar gate electrode is placed in 3000 *μ*m distance.

In devices without source-drain electrode insulation, source and drain electrodes are directly in contact with the water droplet like the gate electrode. Due to absence of insulator overlap with channel area, series parasitic resistance *R*_px_ does not exist, which is desirable for proper transistor operation. For these devices, difference between simulation results of probe- and planar-gate setups is more significant. In planar-gate setups, effect of gate electrode on voltage distribution is drastically lower and *V*_g_EDL_ curves dissociate more for different *V*_DS_ values, compared to probe-gate setup, as seen in Fig. 2c.

The effective voltage on the EDL insulation layer for the WG-FET device is not uniform throughout the channel when *V*_DS_ is not equal to 0 V. Instead, it is a function of the channel position as *V*_g_EDL_ (*x*) as indicated in equation (3). By making linear approximations in simulation results, parameters *k*_1_, *k*_2_, and *k*_3_ are calculated for all WG-FET topologies as given in Table 1.

**Table 1:** Topology specific parameters.

**WG-FET Topology**	**k**_**1**_	**k**_**2**_	**k**_**3**_
Probe Gate with S/D insulation	0.515	0.017	0.021
Planar Gate with S/D insulation (600 *μ*m)	0.454	0.021	0.019
Planar Gate with S/D insulation (3000 *μ*m)	0.315	0.032	0.020
Probe Gate without S/D insulation	0.450	0.026	0.063
Planar Gate without S/D insulation (600 *μ*m)	0.198	0.050	0.071
Planar Gate without S/D insulation (3000 *μ*m)	0.150	0.056	0.073

In probe-gate setup with source-drain electrode insulation, approximately 51.5 % of the applied *V*_GS_ and 1.7 % of the applied *V*_DS_ are transferred as effective gate voltage. Planar-gate setup with 600 *μ*m gate distance and source-drain electrode insulation, has inferior *k*_1_ and *k*_2_ values relative to probe-gate setup. When the gate electrode distance is increased to 3000 *μ*m for the same setup, *k*_1_ decreases to 0.315 while *k*_2_ increases to 0.032, which indicates that the effect of gate electrode weakens with increasing planar gate electrode distance. For devices without source-drain electrode insulation, probe-gate setup has a comparable *k*_1_ value with a slightly elevated *k*_2_ value with respect to devices with source-drain electrode insulation. On the other hand, planar-gate setups have considerably poor *k*_1_ and *k*_2_ values. For planar-gate setup with 600 *μ*m gate distance, *k*_1_ value is halved whereas *k*_2_ value is doubled compared to probe-gate setup. When the gate distance is increased to 3000 *μ*m, they deteriorate further. For planar-gate setups without source-drain electrode insulation, *k*_1_ and *k*_2_ values are comparable as seen in Table 1. This implies that the effect of gate electrode voltage is no longer dominant on *V*_g_EDL_ and the effect of drain electrode gets significant which is not desired for a proper transistor operation. This points to the significance of source-drain electrode insulation on planar gate devices.

### Experimental Results

**Figure 3:**
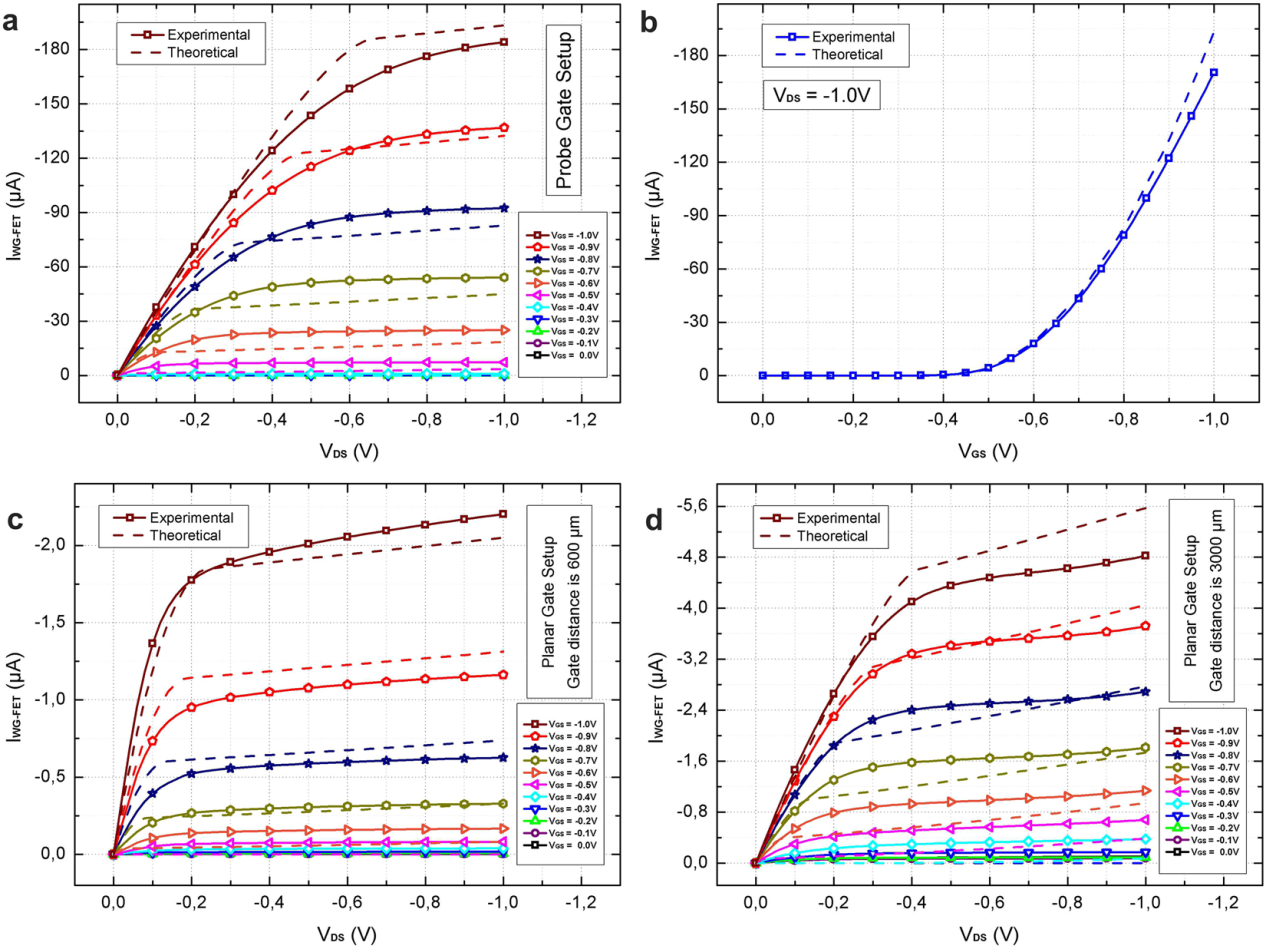
Measurement results for WG-FET devices with source-drain electrode insulation. (**a**) I_DS_–V_DS_ curves for probe gate setup for V_GS_ values swept from 0 V to −1 V. (**b**) I_DS_–V_GS_ curve for the same setup for V_DS_ equals to −1 V. I_DS_–V_DS_ measurement results are also given for planar gate setups with (**c**) 600 μm and (**d**) 3000 μm gate distances. Solid lines show experimental results whereas dashed lines indicate corresponding HSPICE simulation results.

All WG-FET topologies given in Table 1 are fabricated for electrical measurements. Each topology is also simulated with HSPICE by using equations (6) and (7) with corresponding *k*_1_, *k*_2_, and *k*_3_ values for comparison. For devices with source-drain electrode insulation, series parasitic resistances due to insulator overlapping, *R*_px_, are added in HSPICE models. In Fig. 3a,b, *I*_DS_–*V*_DS_ and *I*_DS_–*V*_GS_ measurement results are given for probe-gate setup with source-drain electrode insulation. *I*_ON_ and *I*_OFF_ currents are measured as 184 *μ*A and 8 nA, respectively, which gives an on/off ratio of 23,000 A/A. Transfer curve measurements for *V*_DS_ = −1 V (Fig. 3b) point to an effective threshold voltage of −0.46 V. This corresponds to *V*_thc_ value of −0.26 V. Both *I*_DS_–*V*_DS_ and *I*_DS_–*V*_GS_ measurement results are parallel with simulations. In experiments, no variation in transistor characteristics was noted due to vertical distance of probe gate electrode as long as it stayed in the water droplet. Also, lateral displacement of probe was tested up to 3 mm and no effect was noticed on measurement results. For planar-gate setup with 600 *μ*m gate distance and source-drain electrode insulation, on/off ratio is found as 1,100 A/A. The effective threshold voltage is calculated as −0.56 V, which results in *V*_thc_ value of −0.27 V. Decrease in on/off ratio is expected due to inferior *k*_1_ and *k*_2_ values. Simulations are in agreement with measurement results as seen in Fig. 3c. Similarly, for planar-gate setup with 3000 *μ*m gate distance and source-drain electrode insulation, on/off ratio is found as 45 A/A and effective threshold voltage is calculated as −0.63 V. For this measurement, *V*_thc_ is found as −0.13 V. Increasing gate electrode distance from 600 *μ*m to 3000 *μ*m, decreases on/off ratio further. Theoretical and experimental results are given in Fig. 3d.

**Figure 4:**
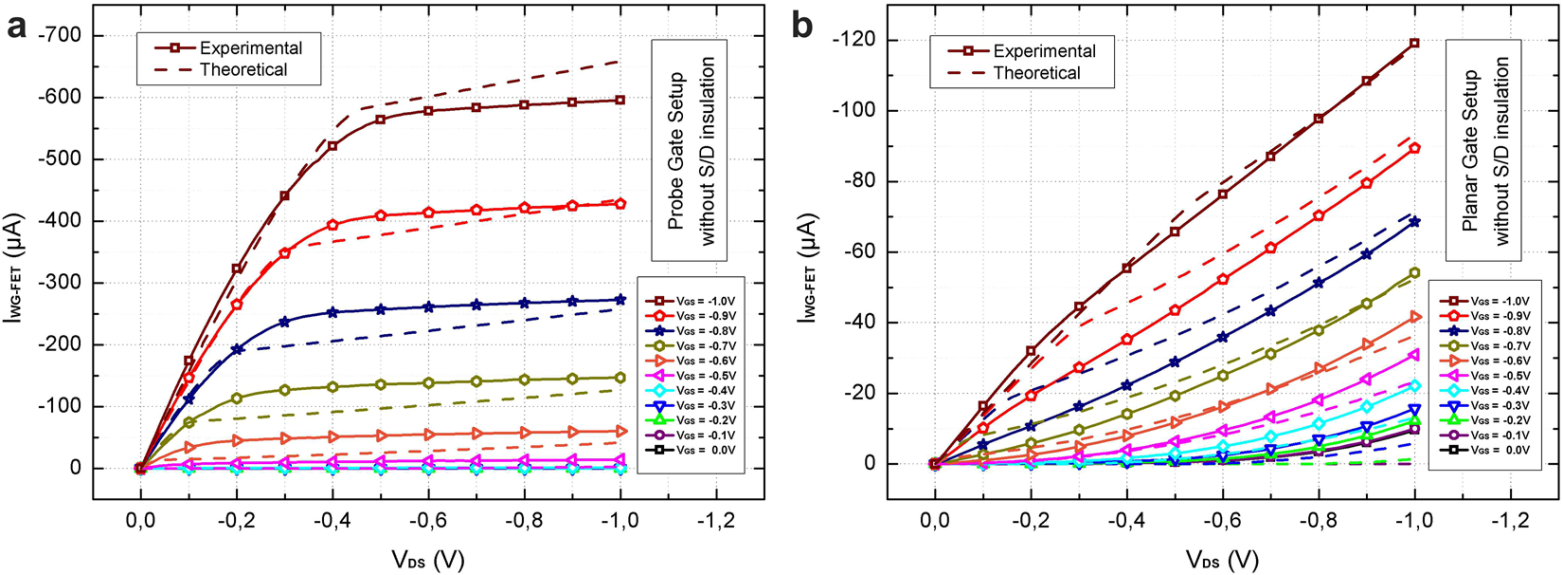
*I*_DS_–*V*_DS_ measurement results for (**a**) probe- and (**b**) planar-gate setups without source-drain electrode insulation. Solid lines show experimental results whereas dashed lines indicate corresponding HSPICE simulation results. *V*_GS_ is swept from 0 V to −1 V.

In Fig. 4a, *I*_DS_–*V*_DS_ measurement results are given for probe-gate setup without source-drain electrode insulation. Inferior *k*_1_ and *k*_2_ values compared to its counterpart with source-drain electrode insulation suggest worse transistor performance. However, *I*_ON_ and *I*_OFF_ currents for this setup are measured as 596 *μ*A and 7 nA, respectively, which gives an on/off ratio of 85,000 A/A. This can be explained due to the absence of *R*_px_. When there is no series parasitic resistance due to insulator overlapping, *I*_ON_ is boosted which significantly increases the on/off ratio. The effective threshold voltage is calculated as −0.51 V, which results in *V*_thc_ value of −0.26 V. Simulation results are also in agreement with measurements as in Fig. 4a. For planar-gate setup without source-drain electrode insulation, *I*_DS_–*V*_DS_ measurement results are given In Fig. 4b. Gate electrode distance is 3000 *μ*m in this layout. Again, absence of *R*_px_ manifests itself with high current levels like in the probe-gate layout. However, deteriorated transistor operation can be seen in this graph as expected from the comparable *k*_1_ and *k*_2_ values. It is hard to mention about a proper threshold voltage or on/off ratio about the planar-gate setup without source-drain electrode insulation. Theoretical simulations demonstrate similar behaviour and support the measurement results as seen in Fig. 4b.

These results show that the best on/off ratio is obtained with probe-gate setup for devices with both insulated and uninsulated source-drain electrodes as expected from *V*_g_EDL_ simulations. Higher *k*_1_ value indicates that higher effective gate voltage can be obtained on the device. In combination with low *k*_2_, a high on/off ratio is expected. For planar-gate topology, the setup with smaller gate distance gives a higher on/off ratio relative to the one with further gate electrode. When the gate distance was increased from 600 *μ*m to 3000 *μ*m, on/off ratio decreased drastically. These findings are in agreement with the device parameters given in Table 1 and supported by the HSPICE simulations. Effects of source-drain electrode insulation are also tested. For probe-gate setup, transistor operation is affected slightly due to uninsulated source-drain electrodes, since it results in a little lower *k*_1_ and a little higher *k*_2_ values as shown in Table 1. However, they result in higher on/off ratio compared to the devices with insulated source-drain electrodes, because these devices do not have series parasitic resistances, *R*_px_. On the other hand, for planar-gate setup without source-drain electrode insulation, *I*_DS_–*V*_DS_ measurement results show considerable deviations from proper transistor operation due to increasing effect of drain voltage on *V*_g_EDL_. Therefore, insulation of source-drain regions is crucial for WG-FET devices with planar-gate topology.

## Discussion

A novel WG-FET device using 16-nm-thick mono-Si film is presented. Devices with probe- and planar-gate topologies, and with and without source-drain electrode insulation are modelled and simulated. Device parameters, *k*_1_ and *k*_2_, model the gating effect of the gate and drain electrodes, respectively. Parameter *k*_3_ has both drain voltage and channel length contributions. As these parameters are extracted from electric field and voltage distribution simulations, current-voltage relations of the transistors are obtained. All modelled and simulated devices are fabricated and tested. Current levels, on/off ratios, and effective threshold voltage values are extracted from measurements and compared with theoretical calculations, verifying them. The threshold constant parameter, *V*_thc_, is found approximately as −0.26 V from the measurements.

Best on/off ratios are obtained with probe-gate setups. 23,000 A/A and 85,000 A/A are measured for devices with insulated and uninsulated source-drain electrodes, respectively. Removing insulation layers on source-drain electrodes in probe-gate devices improves overall device performance even though it results in inferior *k*_1_ and *k*_2_ values. This is because it eliminates a more important factor, the serial resistances under the insulated regions as a result of inevitable alignment errors. In probe-gate devices, lateral or vertical displacement of gate electrode does not result in a significant variation in transistor operation.

Gate electrode distance and source-drain insulation are very critical in planar-gate devices. Their already low on/off ratio decreases from 1,100 A/A to 45 A/A when gate distance is increased from 600 *µ*m to 3000 *μ*m in devices with source-drain electrode insulation. Planar-gate devices with uninsulated source-drain electrodes do not have any proper transistor characteristics. Therefore, insulation of source-drain electrodes is essential for planar-gate setups.

WG-FET is a promising device especially for microfluidic applications. Si/water interface, transistor behaviour and easy fabrication make it suitable to implement and integrate sensors and read-out circuits together.

## Methods

### WG-FET Fabrication

WG-FET devices with probe- and planar-gate topologies are fabricated with a three-mask photolithographic process. First, 16-nm-thick single crystalline Si layer is patterned as active layer on top of 145-nm-thick buried oxide layer. Trilogy etch (126 HNO_3_:60 H_2_O:5 NH_4_F) is used to pattern Si after a lithographic step. Then, Al layer is thermally evaporated on top with approximately 200 nm thickness. After deposition of Al, a second photolithographic step and wet etch are used to pattern source, drain and planar gate electrodes. To obtain ohmic contacts between Al electrodes and Si layer, device samples are thermally annealed at 475 °C for 5 minutes. Annealing process is done under continuous nitrogen flow to prevent any unwanted surface deposition.

A PR layer with 4 *μ*m thickness is used for both source-drain and field oxide insulation. Thermal SiO_2_ thin films are known for having charge traps on interface surface when in contact with water molecules^31^, therefore it is important to insulate field oxide regions to avoid undesired effects of trapped surface charges around the Si active area, and obtain more repeatable results (see Supplementary Material).

**Figure 5:**
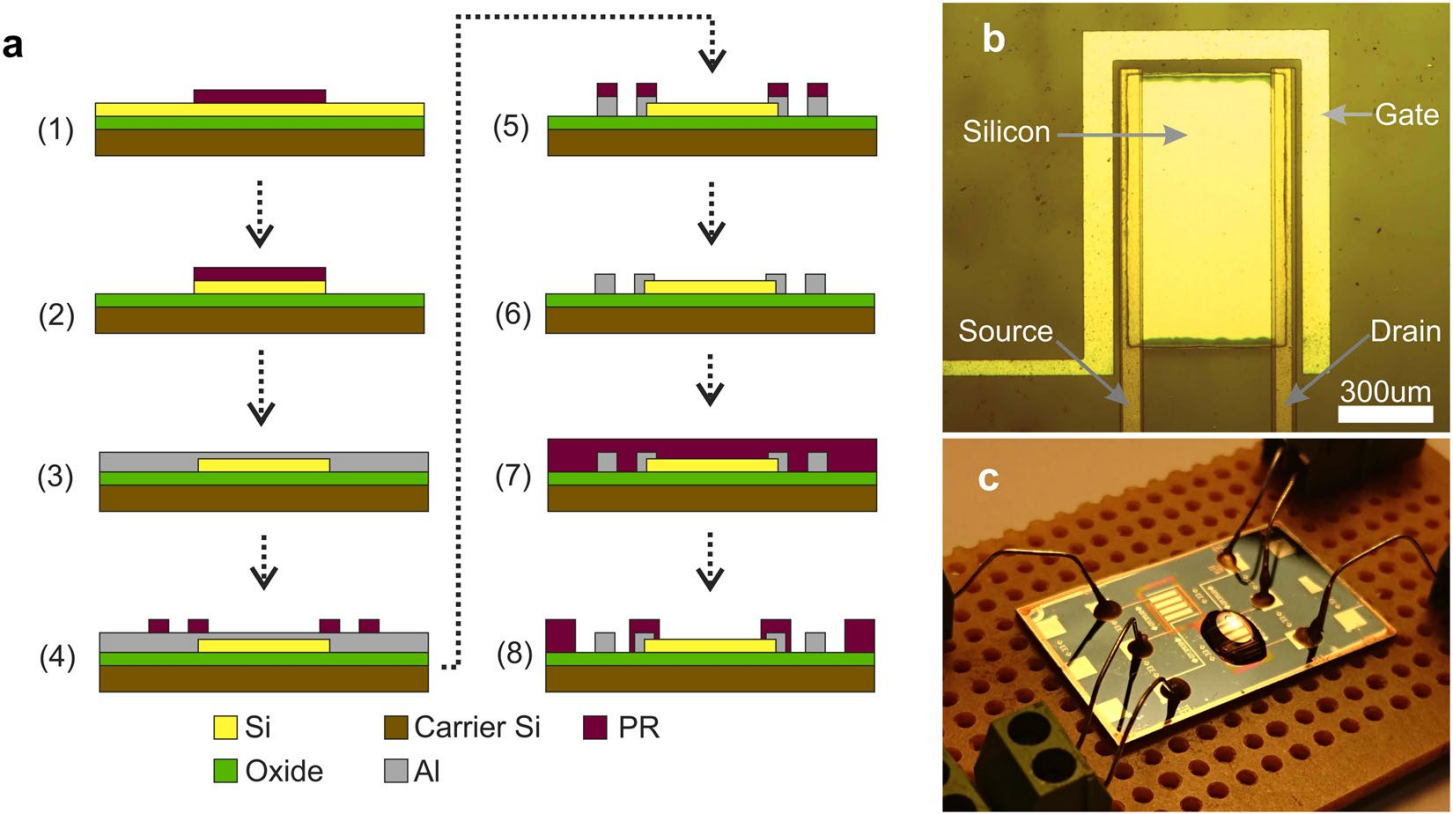
(**a**) Simplified fabrication steps: 1 - PR mask is patterned for Si etch. 2 - Active Si areas are patterned. 3 - PR is stripped and 200 nm Al layer is deposited. 4 - PR mask is patterned for source, drain and gate electrodes. 5 - Al layer is etched and electrodes are formed. 6 - PR mask is stripped and sample is annealed to obtain ohmic contacts between Si layer and source-drain electrodes. 7 - PR layer spin-coated on top. 8 - Insulation layer is patterned. (**b**) A micrograph of a fabricated WG-FET device with planar-gate topology. (**c**) Experimental setup for current-voltage measurements.

Electrical contacts are established with source, drain and planar gate electrodes using silver epoxy. Then, a de-ionized water droplet is placed on top covering both planar gate electrode and the Si active region to complete the WG-FET device. Fabrication steps are summarized in Fig. 5a. A micrograph of a fabricated sample device and a picture of the experimental setup are given in Fig. 5b,c, respectively.

### Experimental Setup

Current-voltage measurements are performed with probe- and planar-gate setups. Probe-gate setup is formed by immersing an external probe into the water droplet just on top of the active Si area. For planar-gate experiments, layouts with electrode distances of 600 *μ*m and 3000 *μ*m are used to examine the effect of gate distance on transistor performance. All devices are tested with and without source-drain electrode insulation for comparison. All measurements are carried out with Keithley 4200SCS semiconductor characterization system. Applied voltage is limited to −1.0 V to prevent electrochemical reaction at electrode/water interfaces.

### Data Availability

The datasets generated during and/or analysed during the current study are available from the corresponding author on reasonable request.

## Electronic supplementary material

Supplementary Information
