# Supplementary material for: Modelling and Realization of a Water-Gated Field Effect Transistor (WG-FET) Using 16-nm-Thick Mono-Si Film
Source: Sci Rep. 2017 Sep 22;7:12190. doi: 10.1038/s41598-017-12439-8 (PMC5610243; doi:10.1038/s41598-017-12439-8)
Supplement: Supplementary file 1 — Supplementary Information [file 41598_2017_12439_MOESM1_ESM.pdf]

Supplementary Information

Modelling and Realization of a Water-Gated Field Effect Transistor (WG-FET) Using 16-nm-Thick Mono-Si Film

Bedri Gurkan Sonmez[1]*, Ozan Ertop*[1]*, and Senol Mutlu*[1,*]

[1] Department of Electrical and Electronics Engineering, Bogazici University, Istanbul, 34342, Turkey

[*] Correspondence and requests for materials should be addressed to S.M.

(E-mail: senol.mutlu@boun.edu.tr)

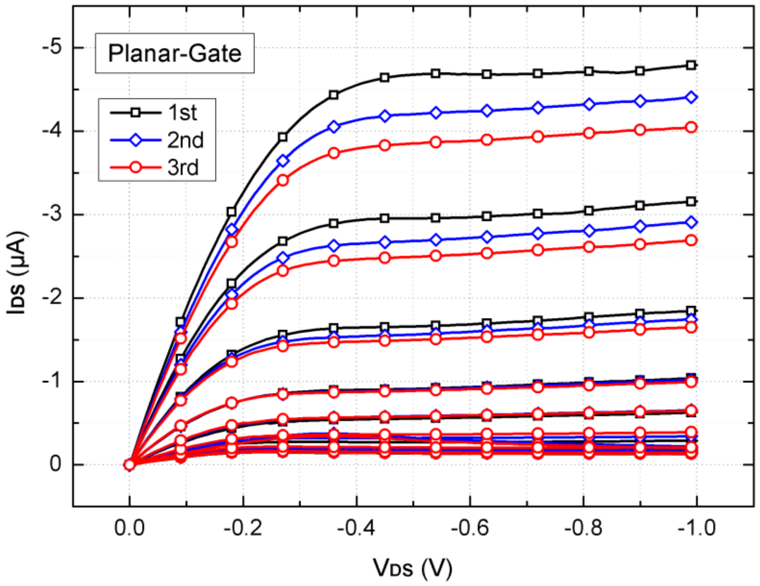

Supplementary Figure S1. Three consecutive measurements with a planar-gate WG-FET device. Measurements are performed with Keithley 4200SCS semiconductor characterization system in "quiet" settings. Each dataset takes 10 minutes to complete, therefore I_{DS}-V_{DS} graph demonstrates a 30 minutes of continuous operation. Only a small degradation in current levels is observed after 30 minutes.

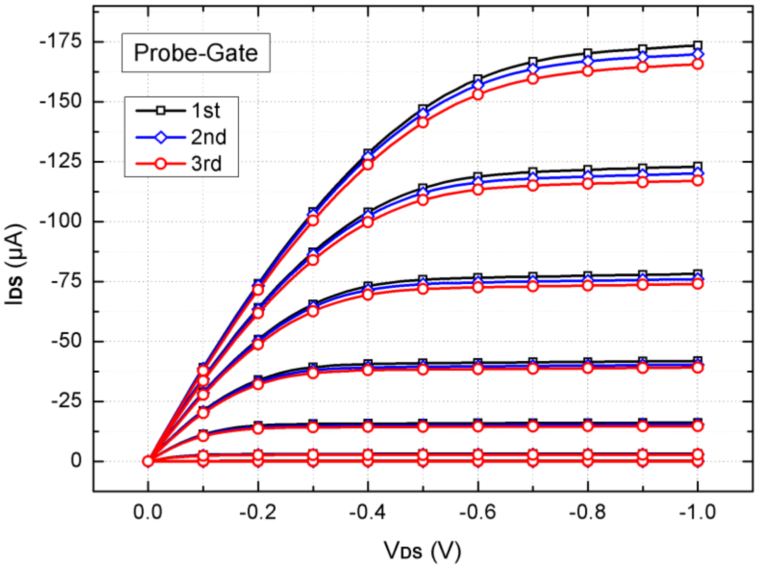

Supplementary Figure S2. Three consecutive measurements with a probe-gate WG-FET device. Similar current levels are obtained even after 30 minutes of operation.

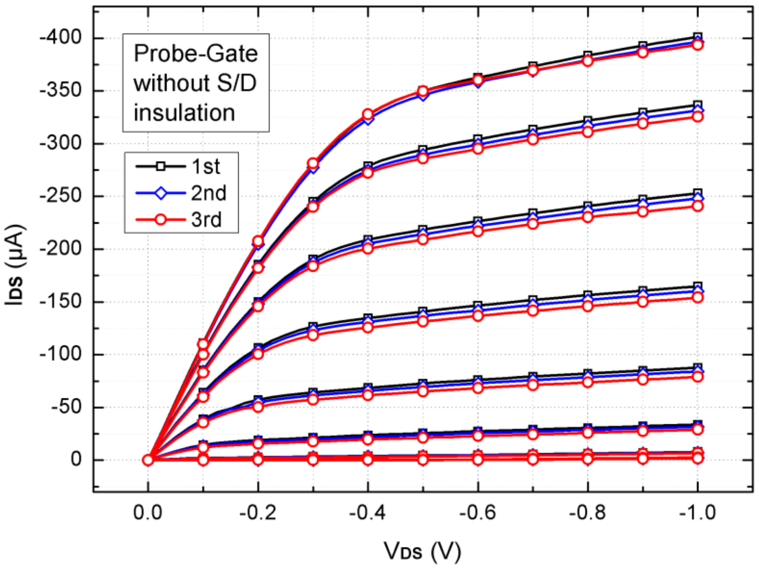

Supplementary Figure S3. Three consecutive measurements with a probe-gate WG-FET device without source-drain electrode insulation. Again, similar current levels are obtained even after 30 minutes of operation.

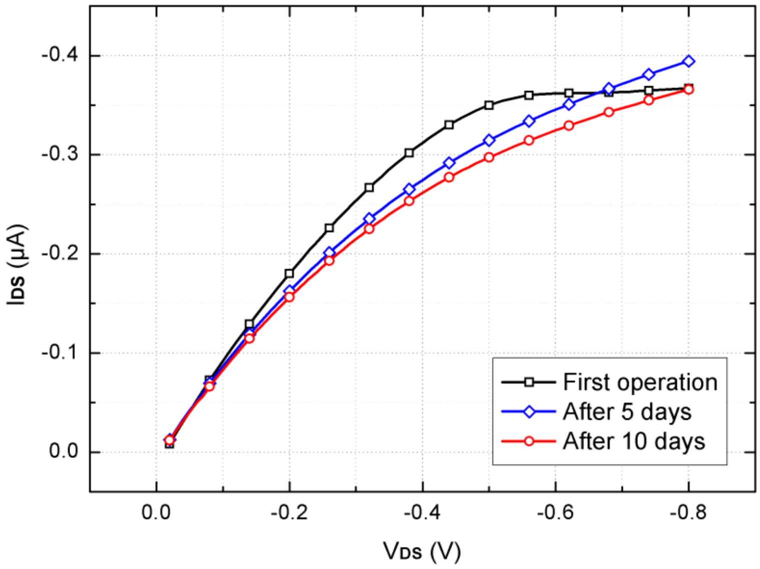

Supplementary Figure S4. Three measurements with a planar-gate WG-FET device with five-day intervals[1]. I_{DS} curves given in the graph are obtained for V_{GS} = -0.8 V. For further testing of repeatability, measurements are repeated with the same device after five and ten days. Every test started with a new water droplet. Similar current levels are obtained at all measurements. Dried WG-FET device is kept in ambient conditions at room temperature during these ten days. No special treatment is applied between measurements. It is observed that although a small degradation is noted within continuous operation, current levels are restored during the non-operating intervals.

References

[1] Sonmez, B. G., Ertop, O. & Mutlu, S. Improved repeatability in planar water-gate field effect transistor (WG-FET) with 16-nm-thick single crystalline Si film. *Procedia Eng.* **168**, 1739-1742 (2016).
